# Concurrent Undisplaced Patellar Fracture and Posterior Cruciate Ligament Avulsion Fracture Managed With Surgical Fixation and Rehabilitation: A Case Report

**DOI:** 10.7759/cureus.64931

**Published:** 2024-07-19

**Authors:** Nikita Gangwani, Pratik Phansopkar

**Affiliations:** 1 Musculoskeletal Physiotherapy, Ravi Nair Physiotherapy College, Datta Meghe Institute of Higher Education and Research (DU), Wardha, IND; 2 Physical Medicine and Rehabilitation, Ravi Nair Physiotherapy College, Datta Meghe Institute of Higher Education and Research (DU), Wardha, IND

**Keywords:** physical therapy, rehabilitation, surgical intervention, combined knee injury, avulsion fracture, posterior cruciate ligament, patellar fracture

## Abstract

This case report explains the successful management of a rare, combined injury: an undisplaced patellar fracture and a posterior cruciate ligament (PCL) avulsion fracture at the tibial attachment in a 44-year-old male patient following a motorbike accident. While both injuries are frequently seen in orthopedic practice, their concurrent occurrence is uncommon. The patient presented with significant knee swelling, limited range of motion, and pain following the accident. An X-ray revealed a patellar fracture and magnetic resonance imaging (MRI) confirmed an undisplaced fracture, a PCL tear, and a medial meniscus injury. The patient underwent surgical intervention for PCL fixation with a cannulated cancellous (CC) screw under spinal anesthesia. Following surgery, a comprehensive rehabilitation program was implemented, focusing on pain management, reducing swelling, regaining range of motion, and strengthening the surrounding musculature. The program progressed through three phases, steadily increasing the intensity and complexity of exercises. The patient exhibited significant improvement in pain, swelling, range of motion, and muscle strength throughout the rehabilitation program. By week 12, he had achieved near-normal knee function and was able to resume most daily activities.

## Introduction

Lower limbs are the most vulnerable anatomical area in trauma patients, sustaining injuries in around 19% of cases. Upper limbs are marginally less affected, with a prevalence of approximately 17.7% [1]. High-impact trauma frequently leads to complex injuries, potentially including isolated or multiple ligament tears in the knee [2]. The patella, the largest sesamoid bone in humans, plays a significant role in the extensor apparatus and articulates with the femur in the patellofemoral joint. The primary function of the patella is to serve as a bridge [3]. Fractures of the patella are frequently seen in orthopedic practice and are extensively documented in the medical literature [4].

The posterior cruciate ligament (PCL) stands out as the most robust ligament within the knee, with injuries being less prevalent compared to those of the anterior cruciate ligament (ACL) [5]. The primary role of the PCL is to prevent excessive backward movement of the tibia toward the femur. The PCL consists of two distinct bundles that work together cooperatively, offering both rotational stability and additional reinforcement. These bundles, known as the anterolateral (AL) and posteromedial (PM) bundles, have different orientations within the knee joint. The AL bundle is present more vertically, while the PM bundle is positioned differently. During different degrees of knee flexion, each bundle plays a specific role; the PM bundle primarily restrains posterior translation during moderate flexion, whereas the AL bundle becomes more active during extension and deeper flexion [6]. PCL avulsion fractures, a distinct subtype of PCL injury, are less frequent than the more common intrasubstance tears of the ligament [7]. The occurrence of both a patellar fracture and an avulsion fracture at the tibial attachment of the PCL is rarely documented in existing literature. Hooper et al. reported that 16.8% of PCL avulsion fractures were associated with meniscal injuries, while 19.1% involved additional ligament injuries. Also, patellar fractures commonly lead to anterior knee pain, which can obscure the diagnosis of a concomitant PCL injury by complicating the performance of the posterior drawer test. Furthermore, avulsion fractures at the tibial attachment of the PCL are frequent occurrences in the practice of orthopedic surgery [8]. However, there is scarce literature about meniscus injuries with PCL ligaments [9].

Fractures of the patella can stem from either direct or indirect causes. An example of the classic indirect mechanism is when someone falls on their feet, prompting the quadriceps to eccentrically contract in an attempt to slow down the body's descent. If the force of the fall surpasses the knee's ability to resist flexion, the extensor mechanism may fail, resulting in a patellar fracture. Similarly, avulsion fractures of the PCL at its tibial insertion often occur due to a specific event known as a dashboard injury. This happens when the knee is in a flexed position, and something hits it directly from the anterior side. The combination of knee flexion and a posteriorly directed force applied to the pretibial area can lead to an avulsion fracture of the PCL at its attachment point on the tibia [10,11]. Failure to address these injuries appropriately can result in a range of complications, including persistent laxity in the posterior knee, loss of motion, ongoing knee pain, fractures, osteonecrosis, compartment syndrome, and the development of heterotopic ossification. Neurovascular injuries such as a popliteal artery, common peroneal nerve, and tibial nerve injuries can also be seen in PCL avulsions [12]. This case report describes a surgical solution for knee joint instability. The procedure involves fixing the PCL with a cannulated cancellous (CC) screw [13]. Following surgery, a tailored rehabilitation program is implemented to guide the patient's recovery in stages [14].

## Case presentation

Patient information

A 44-year-old male patient presented with significant knee swelling and limited range of motion following a road traffic accident on January 7, 2024. The accident occurred while he was traveling on a motorcycle to his hometown when he encountered the sudden appearance of a truck from the opposite direction. To avoid a collision, he swerved, resulting in a crash with a divider. Although he fell with his knee flexed, he remained conscious without any bleeding from the ear, nose, or throat, nor did he experience nausea or vomiting. Upon initial assessment at a nearby hospital, an X-ray conducted on January 8, 2024, revealed a patellar fracture, causing severe pain and swelling in his right knee, especially during knee flexion. Subsequently, he was referred to our hospital for further evaluation, where a magnetic resonance imaging (MRI) on January 10, 2024, revealed a minimally displaced patellar fracture, a tear in the PCL, and injury to the posterior horn of the medial meniscus. Until January 15, 2024, conservative management with a long plaster cast was implemented. On January 17, 2024, the patient underwent surgical intervention for PCL fixation with a 4.5 mm CC screw under spinal anesthesia.

Clinical examination

Before the examination began, we obtained the patient's informed consent for the procedure and explained the possibility of publishing their case as a report. The patient was alert and cooperative and demonstrated full orientation to their surroundings, including time, location, and personal identity. The patient was seen supine, lying with the right leg elevated with the help of a long pillow. A patellar tendon-bearing cast was present to manage the patellar fracture conservatively. This assessment was taken before his surgery; on observation, the patient exhibited a mesomorphic physique with no signs of edema or muscle atrophy. The patient's present pain magnitude was measured with a visual analog scale (VAS) of 7.5 which indicates severe pain at the knee joint during palpation; the patient displayed grade 4 tenderness in specific areas, including the medial aspect of the right thigh. The skin was noted to be dry, with swelling evident over the right thigh and medial aspect of the distal thigh and around the popliteal fossa of the right lower limb. The skin was noted to be dry, with swelling evident over the right thigh and medial aspect. On examination, his ranges were taken with the help of a goniometer, and manual muscle testing was done.

Table 1 depicts the timeline of events for the patient.

**Table 1:** Timeline of key events and clinical interventions. PCL: Posterior cruciate ligament; CC: Cannulated cancellous; MRI: Magnetic resonance imaging

Date	Event	Description
January 7, 2024	Date of motorbike accident	The patient was involved in a motorbike accident, sustaining a knee injury.
January 8, 2024	X-ray was taken	X-ray confirms patellar fracture; patient experiences severe pain and swelling in the knee.
January 10, 2024	The patient was shifted to our hospital for further investigation, such as an MRI.	An MRI reveals an undisplaced patellar fracture, PCL tear, and meniscal injury, and the patient begins conservative management with a patellar tendon-bearing cast for a knee injury.
January 15, 2024	Surgery was performed	Open reduction and internal fixation of the PCL avulsion fracture on the on the right side with the CC screw.
January 17, 2024	The physiotherapy session started	A comprehensive, tailor-made protocol was given for about 12 weeks.

Investigations

Before undergoing surgery, the patient underwent several investigations to assess their overall health status and evaluate the extent of knee injuries. These investigations included a complete blood count (CBC), kidney function test (KFT), and liver function test (LFT), all of which returned within normal ranges, indicating no underlying systemic abnormalities.

Furthermore, pre-operative imaging studies were conducted to precisely assess the extent of knee injuries. The X-ray revealed a non-comminuted transverse patellar fracture, indicating a fracture across the patella without fragmentation. Additionally, the pre-operative MRI provided detailed insights, revealing a complete tear of the PCL, a grade 2 tear of the posterior horn of the medial meniscus, and an undisplaced fracture of the patella. These diagnostic findings were crucial in guiding the surgical approach and planning the appropriate interventions to address the identified injuries comprehensively.

**Figure 1:**
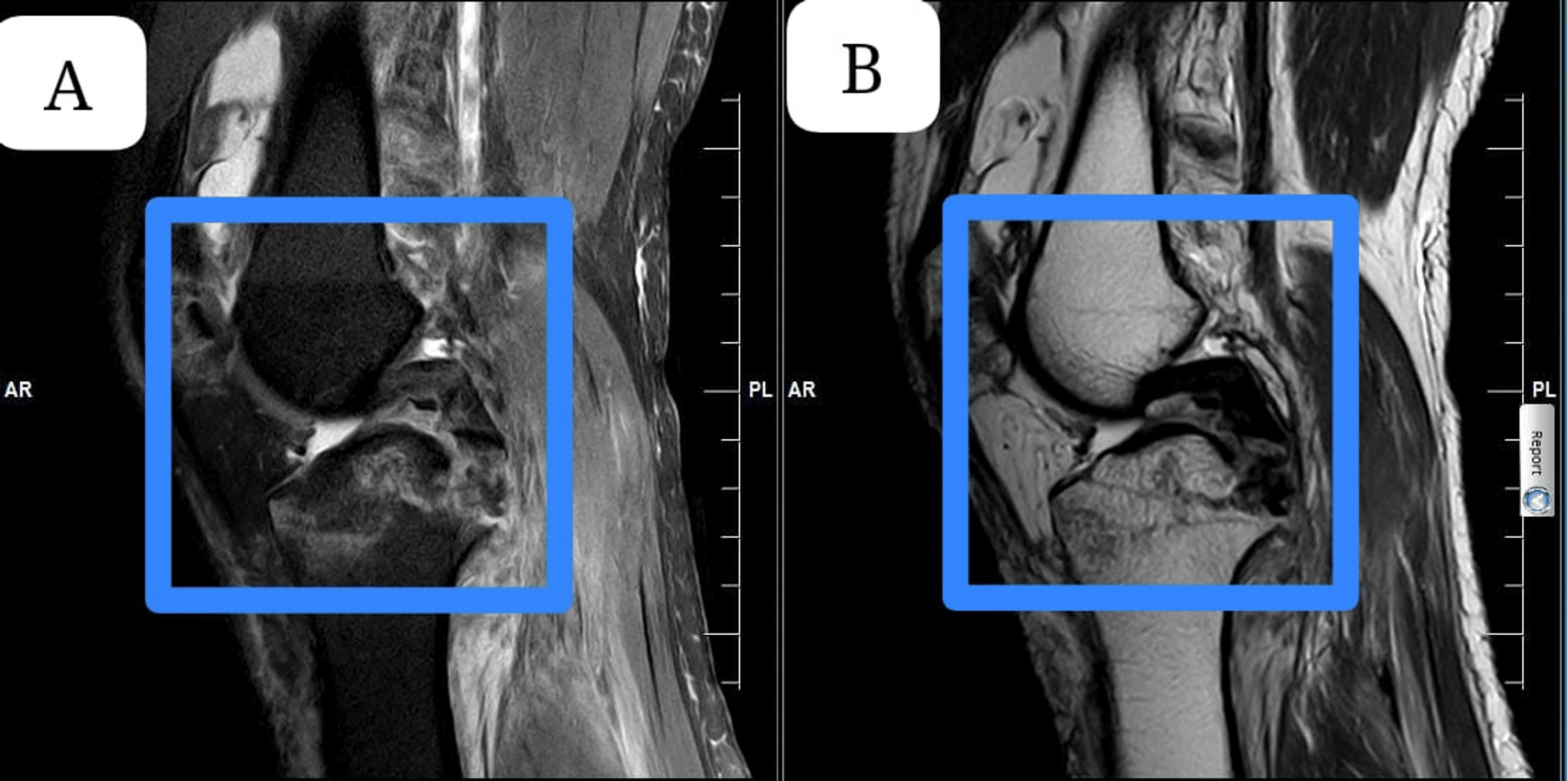
The MRI scans show a posterior cruciate ligament (PCL) injury. Panel A-B: Preoperative MRI scan of the knee showing the affected area. The highlighted region indicates abnormalities in the joint structure.

In Figure 1, a recent MRI analysis of the knee shows a PCL injury accompanied by bone marrow edema. The joint space and cartilage appeared normal, and there were evident meniscal tears.

Surgery details

On the surgical table, the patient was placed in a prone posture with precautionary padding to shield bone prominences. The skin was carefully incised into a 5 cm S shape, and the muscles between the gastrocnemius and semimembranosus were then carefully dissected apart. The next procedure was a vertical capsular incision. Evaluation of the best fixing technique was made possible by the identification of skeletal pieces. In order to secure the fracture reduction, two Kirschner wires were inserted into the tibia. A 4.5-mm CC screw was placed along the posterior portion of the tibial intercondylar eminence to complete the stabilization once reduction was verified. Using C-arm imaging, the appropriate decrease was confirmed. The surgical wound was carefully sutured shut after it had been well-irrigated. After a smooth and effective surgery, the patient was moved to the intensive care unit for recovery.

**Figure 2:**
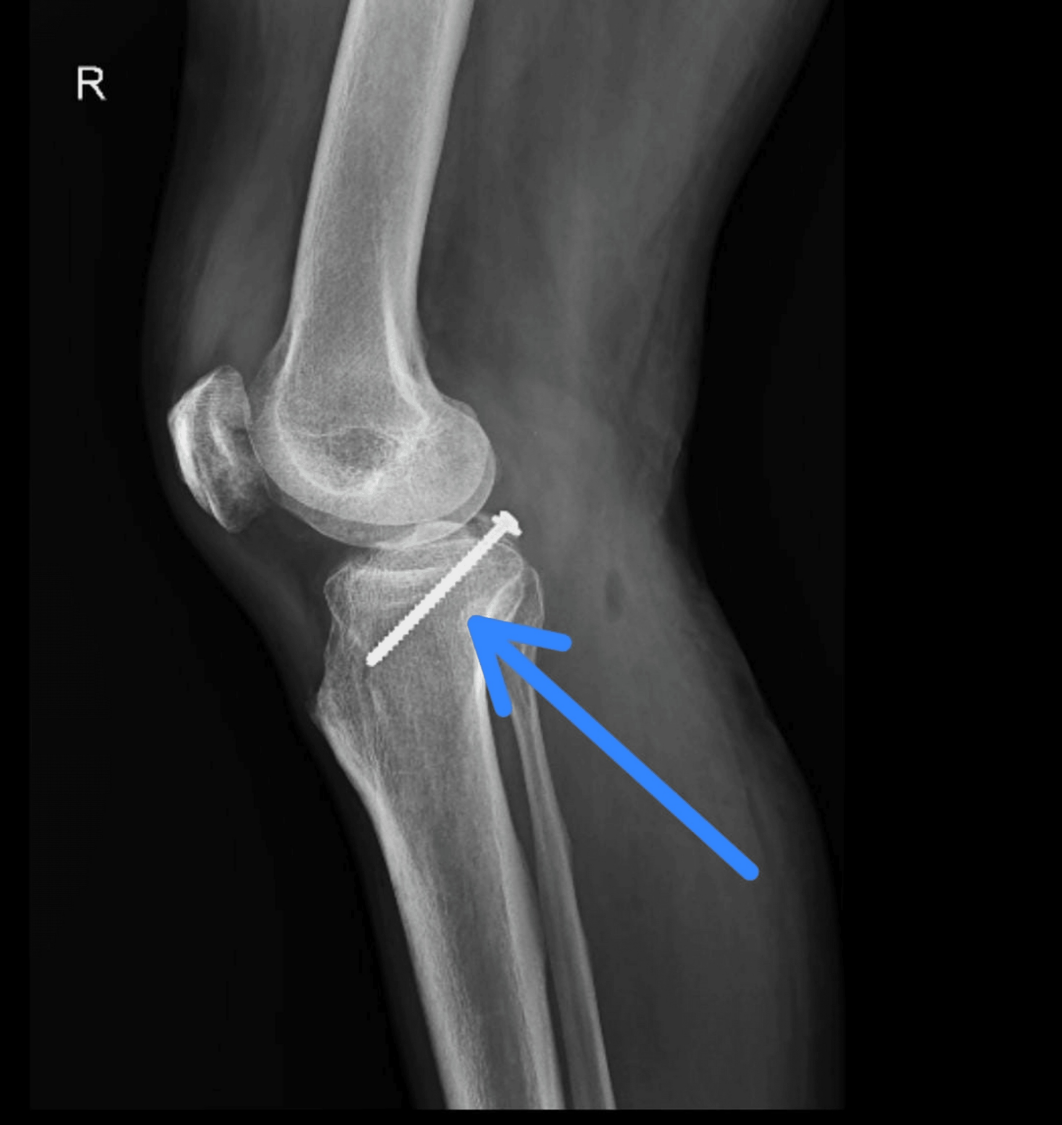
A lateral view of the X-ray shows a right knee with a screw used to fix an avulsion of the posterior cruciate ligament (PCL).

**Figure 3:**
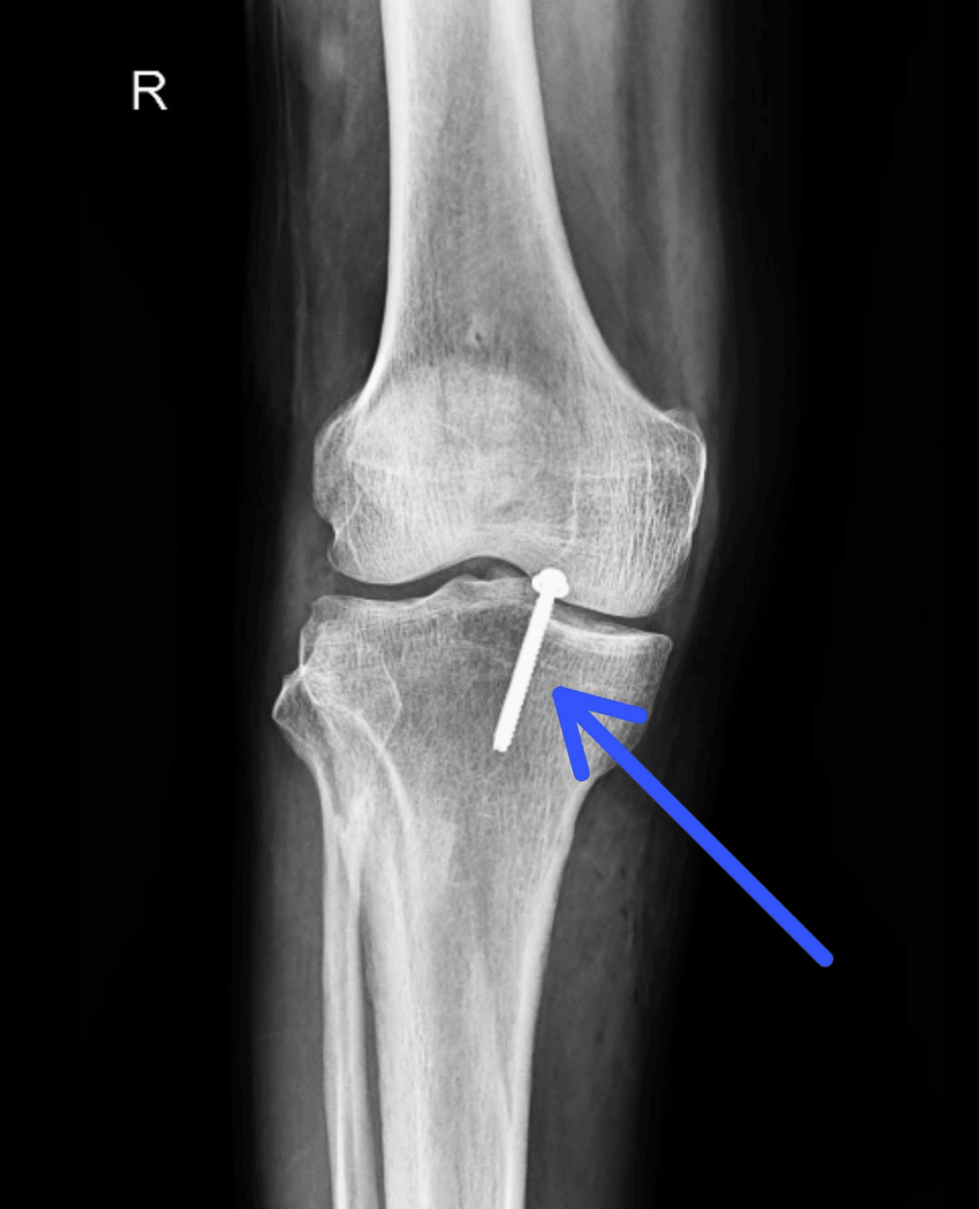
Anteroposterior (AP) view of the X-ray shows a right knee with a screw used to fix an avulsion of the posterior cruciate ligament (PCL).

Figures 2, 3 show a postoperative X-ray of the right knee showing PCL avulsion fixation in the lateral and anteroposterior (AP).

Diagnostic assessment

On postoperative day one, following knee surgery, patients typically experience severe pain, with VAS scores ranging around 7.8 and noticeable swelling around the knee. Initial assessments focus on measuring the range of motion (ROM) in knee flexion and extension, which is often limited due to pain and swelling, and evaluating muscle strength through manual muscle testing (MMT), which typically shows reduced strength. MMT for the hip in the pre-operative state, for the hip and knee was assessed in a side-lying position, and for the ankle in the prone position. MMT in the postoperative state is checked in sitting and side-lying for the hip, prone for the knee, and standing for the ankle. Functional outcome measures involve the knee injury and osteoarthritis outcome score (KOOS), assessing pain, symptoms, daily living activities, sports function, and quality of life; the timed up and test (TUG), evaluating basic functional mobility and balance; and the lower extremity functional scale (LEFS), determining the patient's ability to perform everyday tasks. These evaluations establish a baseline for guiding the initial stages of rehabilitation and monitoring recovery progress. Table 2 presents the active ROM measurements. 

**Table 2:** Active range of motion measurements for the right hip, knee, and ankle pre-operatively, on postoperative day two/pre-rehabilitation day one, post-rehabilitation week six, and post-rehabilitation week 12.

Joint and Movements (Right side)	Pre-operative	Postoperative Day 2/Pre-Rehabilitation Day 1	Post rehabilitation (week 6)	Post rehabilitation (week 12)
Hip				
Flexion	0-10°	0-20°	0-60°	0-110°
Extension	0-5°	0-10°	0-20°	0-30°
Abduction	0-20°	0-30°	0-40°	0-45°
Adduction	0-10°	0-15°	0-30°	0-45°
Internal Rotation	0-5°	0-10°	0-20°	0-25°
External Rotation	0-5°	0-10°	0-20°	0-25°
Knee				
Flexion	0-35°	0-20°	0-70°	0-120°
Extension	35°-0	20°-0	70°-0	120°-0
Ankle				
Dorsiflexion	0-5°	0-5°	0-10°	0-35°
Plantarflexion	0-10°	0-10°	0-20°	0-20°

Table 3 details the manual muscle testing (MMT) scores for the same joints and movements across the same time points.

**Table 3:** Manual muscle testing (MMT) scores for the right hip, knee, and ankle pre-operatively, on postoperative day two/pre-rehabilitation day one, post-rehabilitation week six, and post-rehabilitation week 12. 0: No movement, no contraction experienced or observed in the muscle; 1: Tendon becomes a protruding or frail contraction experienced in muscle with no visible movement; 2: Movement through a partial range of motion; 2: Movement through a complete range of motion for the muscle being tested; 2+: Holds against slight pressure in the test position; moves through a partial range of motion against gravity; 3: Gradual release from the test position occurs; 3: Holds the test position (with no added pressure); 3+: Holds test position against minor pressure; 4: Holds test position against minor to moderate pressure; 4: Holds test position against moderate pressure; 4+: Holds test position against moderate to strong pressure; 5: Holds the test position against strong pressure/normal

Joint and movements (Right side)	Pre-operative	Postoperative Day 2/Pre-Rehabilitation Day 1	Post rehabilitation (week 6)	Post rehabilitation (week 12)
Hip				
Flexion	2/5	3/5	5/5	5/5
Extension	2/5	3/5	5/5	5/5
Abduction	2/5	3/5	5/5	5/5
Adduction	2/5	3/5	5/5	5/5
Internal rotation	2/5	2/5	3+/5	5/5
External rotation	2/5	2/5	3+/5	5/5
Knee				
Flexion	2/5	1/5	3+/5	5/5
Extension	2/5	1/5	3+/5	5/5
Ankle				
Dorsi flexion	3/5	3/5	3+/5	5/5
Plantar flexion	3/5	3/5	3+/5	5/5

Physiotherapeutic intervention

After PCL avulsion, potential complications like knee hyperextension and tibial translation necessitate early post-surgery physiotherapy. The treatment targets swelling, joint effusion, knee pain, and limited motion. Weekly rehabilitation progresses from managing swelling to restoring motion and strength. Cryotherapy, compression, and elevation alleviate initial inflammation. Subsequent weeks focus on regaining full knee extension and flexion through passive and active exercises, emphasizing proper alignment. Strengthening exercises target muscles around the knee, starting with isometrics and advancing to dynamic exercises. Pain monitoring and exercise progression prevent overexertion. Manual therapy may address residual stiffness. This tailored rehabilitation approach minimizes complications and promotes joint function, strength, and stability post-PCL reconstruction and open reduction internal fixation (ORIF) with a locked patella nail for the patella.

This protocol is made with a combination of different exercises and protocols taken from therapeutic exercise foundations and techniques by Kisner, Carolyn, and Colby [15] and Massachusetts General Brigham Sports Medicine [16]. The physiotherapy intervention protocol with rationale and description is mentioned in Tables 4-6.

**Table 4:** Physiotherapy intervention in phase 1, maximum protection phase.

Phase 1	Description	Rationale
Goals	Pain relief	Alleviating pain, promoting resorption, improving joint mobility, and preventing functional/structural damage are essential to enhancing comfort, expediting healing, restoring mobility, and ensuring long-term joint health and functionality.
	Promoting resorption	
	Improving joint mobility	
	Avoiding functional and structural damage	
Patient Education	1. Follow post-operative instructions precisely, including medication schedules and activity limitations.	Adherence to post-operative instructions ensures optimal healing and reduces complications, while lifestyle modifications support recovery by promoting healthy habits. Wearing an immobilizer during the day prevents tibial displacement, safeguarding the surgical site, and gradual mobility resumption aids in restoring function without undue stress on healing tissues.
	2. Make lifestyle modifications as recommended to support your recovery. To help your recovery, consume a balanced diet rich in calcium and vitamin D to promote bone healing. Avoid strenuous activities that could stress the knee joint and elevate your injured leg above your heart whenever possible to reduce swelling.	
	3. Wear an immobilizer during the day to prevent posterior displacement of the tibia.	
	4. Gradually resume mobility.	
Precautions	1. Protective bracing: Employ a knee brace during weight-bearing activities for ligament protection.	Utilizing a knee brace provides structural support, reducing the risk of ligament strain during weight-bearing, while gradual activity progression minimizes stress on healing tissues, promoting optimal recovery. Monitoring for complications and avoiding twisting motions mitigate potential disruptions to the healing process, ensuring favorable outcomes.
	2. Gradual progression: Slowly advance activities as directed by healthcare professionals to prevent setbacks.	
	3. Monitor for complications: Stay vigilant for pain, swelling, instability, or restricted motion; report promptly.	
	4. Avoid twisting movements: Minimize twisting or pivoting motions to safeguard the healing ligament.	
	5. Avoid repetitive flexion of the knee: During the rehabilitation process, restrict the frequency of knee flexion exercises to reduce the risk of irritating the PCL graft.	
Intervention	1. Cryotherapy, compression, and elevation: Apply cold packs and compression bandages and elevate the affected limb to reduce swelling and promote healing.	1. Cryotherapy reduces inflammation and pain, compression minimizes swelling, and elevation promotes fluid drainage.
	2. Ankle pumping exercise: Perform controlled dorsiflexion and plantar flexion movements of the ankle to enhance blood circulation and prevent venous stasis. [10x3 sets].	2. Ankle pumping improves blood circulation and reduces the risk of blood clots.
	3. Voluntary muscle activation: Engage in isometric contractions followed by dynamic movements of the knee and hip muscles to improve strength and range of motion. [10x3sets with holds of 5-10 seconds].	3. Voluntary muscle activation strengthens muscles and improves the range of motion, which is essential for regaining stability and function.
	4. Active-assisted range of motion (A-AROM) for knee flexion/extension: Assist the patient in actively moving the knee through its range of motion while providing support as needed. [10x3sets].	4. A-AROM for knee flexion/extension assists the patient in regaining knee movement while providing support to prevent injury.
	5. Multi-angle straight leg raise (SLR): Lie on the back and lift the leg straight up, alternating between different angles to strengthen the hip flexors and quadriceps. [10x3 sets].	5. Multi-angle SLR strengthens the hip flexors and quadriceps, crucial muscles for walking and stair climbing.
	6. Low-intensity quadriceps isometrics: Perform static contractions of the quadriceps muscle at various knee angles to improve muscle activation and strength. [10x3sets with holds of 5-10 seconds].	6. Low-intensity quadriceps isometrics improve muscle activation and strength without putting stress on the joint.
	7. Electrical muscle stimulation (EMS): A surged faradic muscle stimulator is used. Electrodes are applied to the quadriceps muscle, and electrical impulses are delivered to induce muscle contractions, aiding muscle activation and strengthening. [For 30 minutes].	7. EMS stimulates muscle contractions, aiding in muscle re-education and strengthening, which is especially beneficial for patients with weak muscles.
	8. Patellar mobility: Include patellar mobilization to prevent adhesions. Maitland mobilization is given in superior, inferior, medial, and lateral glides.	8. Patellar mobilization prevents scar tissue formation and improves kneecap mobility, reducing pain and stiffness.
	9. Ambulation: Initiate with non-weight bearing for 2 weeks with a walker, slowly progress to partial weight bearing after 2 weeks with crutches. (with a protective brace).	9. Ambulation with weight progression strengthens leg muscles, improves balance, and prepares the patient for walking independently.
	10. Closed chain exercise: After 2 weeks, initiate with- bilateral weight shifting, mini squats to 10º-30º, and spot marching (10x3 sets).	10. Close-chain exercises (bilateral weight shifting, mini squats, spot marching) mimic functional activities and improve strength, balance, and coordination for everyday tasks.
Progression	1. Minimal joint swelling: Strive for diminished intra-articular inflammation.	Reducing joint swelling helps ease discomfort and promotes rehabilitation progress. Fully extending the knee restores normal movement and improves mobility. Having a flexible knee range of 60-70 degrees supports daily activities. Strengthening the quadriceps and hamstrings to a moderate level enhances joint stability, lowers injury risk, and improves function.
	2. Full, active knee extension: Attain complete knee extension through voluntary effort.	
	3. 60-70° knee flexion: Achieve a flexion range of 60-70 degrees for optimal joint mobility.	
	4. Grade 3/5 quadriceps and hamstring strength: Aim for substantial muscle strength enhancement, graded at level 3 out of 5 on manual assessment.	

**Table 5:** Physiotherapy intervention in phase 2, moderate protection phase. PCL: Posterior cruciate ligament; ORIF: Open reduction internal fixation

Phase 2	Description	Rationale
Goals	1. Focus on regaining complete knee extension and flexion through targeted exercises and consistent therapy.	The goals prioritize restoring full knee mobility, enhancing muscle strength and stability, improving gait patterns and motor control, and rebuilding cardiovascular fitness to support comprehensive rehabilitation and promote optimal recovery post-PCL reconstruction and ORIF for patellar fracture.
	2. Implement a structured strengthening program to improve muscle power, joint stability, and endurance.	
	3. Work on gait training and neuromuscular exercises to restore proper walking patterns and motor control.	
	4. Incorporate cardiovascular exercises to rebuild overall fitness and support the recovery process.	
Patient education	1. Avoid high-risk activities such as running, jumping, or heavy lifting that could jeopardize your recovery.	These guidelines aim to protect the healing process post-PCL reconstruction and ORIF for patellar fractures by minimizing stress on the knee joint. Avoiding high-risk activities, utilizing assistive devices, and gradually transitioning to weight-bearing activities support safe and effective recovery, reducing the risk of complications and promoting optimal healing.
	2. Use assistive devices to help with daily living activities if needed. Avoid putting undue stress on your knees when moving around the house.	
	3. Avoid deep knee bends or high-impact activities.	
	4. Begin transitioning from partial weight-bearing to full weight-bearing activities, always using your assistive device and brace as recommended.	
Precautions	1. Controlled knee movements: Focus on exercises that control knee motion within a safe range, avoiding deep knee bends and high-impact activities.	These guidelines are designed to optimize recovery and minimize the risk of complications following PCL reconstruction. Controlled knee movements, focusing on exercises within a safe range, prevent excessive strain on the healing knee joint, reducing the likelihood of setbacks. Continued use of a knee brace provides stability and protection, safeguarding against excessive movements that could disrupt healing. The gradual progression of weight-bearing activities under healthcare provider guidance ensures controlled loading of the knee joint, minimizing the risk of stress or strain. Modification or avoidance of hamstring exercises reduces stress on the PCL graft, which is crucial for graft healing and the prevention of re-injury. Avoidance of knee hyperextension in exercises and daily activities prevents excessive strain on healing tissues and supports proper alignment, promoting effective healing. By adhering to these guidelines, individuals undergoing PCL reconstruction can support a safe and successful recovery process.
	2. Use of knee braces: Continue wearing a knee brace as prescribed to protect the knee from excessive movements and provide stability.	
	3. Gradual weight bearing: Progress weight-bearing activities slowly and as directed by your healthcare provider, using crutches or a walker as needed.	
	4. Hamstring exercise modification: Modify or avoid exercises that heavily engage the hamstrings to prevent stress on the PCL graft.	
	5. Avoid hyperextension: Ensure all exercises and daily activities avoid hyperextending the knee. Use support or assistance when necessary.	
Intervention	1. Range of motion and joint mobility exercises:	
	Continue low-intensity, end-range self-stretching of quadriceps in the standing quads stretch, kneeling hip flexor stretch, standing gastrocnemius stretch, and soleus stretches to gain full knee range. Perform 3 sets of each stretch, holding for 30 seconds.	These stretches (quadriceps, hip flexor, and calf) improve knee flexibility by targeting tightness and promoting balanced muscles.
	Grade 3 mobilization techniques for patellar mobilization to restore full knee flexion. [in superior/inferior and medial/lateral glide].	Patellar mobilization (superior/inferior & medial/lateral glides) prevents stiffness and ensures smooth kneecap movement by preventing adhesions and maintaining proper alignment. This is key to regaining full knee flexion.
	2. Strength training:	
	Initiate with open-chain exercises such as quadriceps strengthening, and straight leg raises with a resistance band. Hamstring strengthening with hamstring curl in prone lying with resistance bands. Hip abductor strengthening with side leg raises with resistance bands calf muscle strengthening with calf raises. Perform 3 sets of 10 repetitions of each exercise. Closed-chain exercises such as bridging and mini squats to 40º-60 step up and step down. Perform 3 sets of 10 repetitions of each exercise.	Open chain exercise: leg raises, hamstring curls, side leg raises, and calf raises (with resistance) target individual muscles to rebuild strength and endurance, protecting the healing joint during recovery. Bridging, mini squats, and step-ups mimic daily movements to strengthen all leg muscles, improve joint stability, and enhance coordination for everyday activities.
	3. Neuromuscular control exercise:	Balance exercises like single-leg stance and progressing to eyes closed, improve balance, proprioception, and neuromuscular control, which are important for preventing falls and ensuring stability. Proprioceptive drills, such as controlled mini squats, enhance feedback and joint stability for safe and effective movement. Perturbation exercises on a foam pad challenge balance and proprioception, promoting adaptive responses and overall strength.
	Balance exercises (e.g., single-leg stance): Hold for 30-60 seconds on each leg, progressing to performing with eyes closed.	
	Proprioceptive drills (e.g., mini squats): Perform 2-3 sets of 10 repetitions, focusing on controlled movements.	
	Perturbation exercises (standing on a foam pad): Hold for 30-60 seconds, challenging balance with small perturbations.	
	4. Core and pelvic strengthening:	
	Glute bridge with holds	
	Glute bridge with abduction/adduction	
	Core strengthening	
	Fire hydrants	
	5. Gait training:	
	Ambulation without brace: Initially, perform ambulation without a brace to promote muscle activation and proprioception.	Ambulation without a brace: Initially, walking without a brace promotes muscle activation and proprioception, encouraging natural movement patterns and muscle engagement.
	Ambulation with a brace: progress to ambulation with a brace for added support and stability, especially during the early stages of rehabilitation.	Ambulation with a brace: Using a brace provides added support and stability, especially during the early stages of rehabilitation when the joint is still vulnerable. It helps prevent undue stress and protects the healing tissue.
	Progression:	
	Start with short distances on level surfaces, gradually progressing to longer distances and varied terrain.	Progression: Gradually increasing distance and terrain complexity ensures a safe transition back to full functional mobility. Emphasizing symmetrical weight-bearing and proper gait mechanics promotes even muscle development and reduces the risk of compensatory injuries.
	Focus on symmetrical weight-bearing, ensuring equal weight distribution between both legs.	
	Emphasize proper step length and timing, coordinating movements of both lower limbs.	
	6. Aerobic conditioning:	
	Stationary cycling: Begin with 10-15 minutes at a low intensity, gradually increasing the duration to 30-45 minutes.	Stationary cycling: Starting with low intensity cycling helps improve cardiovascular fitness, promote blood circulation, and enhance joint mobility without placing excessive stress on the knee. Gradually increasing duration aids in building endurance.
	Brisk walking: Start with 15 minutes, increasing to 30-45 minutes at a moderate intensity.	Brisk Walking: Beginning with shorter durations and gradually increasing to longer, moderate intensity walking sessions improve cardiovascular health, support weight management, and enhance overall endurance and functional mobility.
Progression	1. The absence of pain and joint effusion is observed.	These criteria mark significant progress in post-operative recovery, indicating successful management of pain and inflammation, restoration of full knee mobility, and substantial muscle strength rehabilitation, essential for optimal function and stability.
	2. Complete attainment of active range of motion in the knee joint is achieved.	
	3. A minimum of 75% strength in the knee musculature is demonstrated relative to the contralateral side.	

**Table 6:** Physiotherapy intervention in phase 3, minimum protection phase. PCL: Posterior cruciate ligament; ORIF: Open reduction internal fixation

Phase 3	Description	Rationale
Goals	1. Continued enhancement of muscular strength.	The outlined rehabilitation goals aim to ensure a comprehensive recovery post-PCL reconstruction and ORIF for a patellar fracture. Firstly, enhancing muscular strength is crucial for joint stability and injury prevention. Secondly, attaining neuromuscular control and agility improves movement patterns and reduces the risk of falls. Lastly, engaging in progressively challenging functional activities bridges the gap between rehabilitation and daily tasks, promoting confidence and optimal function. These goals collectively address diverse needs, facilitating a successful return to normal activities with minimized complications.
	2. Attainment of neuromuscular control and agility.	
	3. Engagement in progressively challenging functional activities.	
Patient education	1. Continued strength Building: Focus on strengthening your quadriceps, hamstrings, and calves for improved stability and function.	The outlined rehabilitation goals are designed to address key aspects crucial for optimal recovery following PCL reconstruction and ORIF for a patellar fracture. Continued strength building is essential to restoring muscle function and joint stability, facilitating a return to normal activities. Improved range of motion aims to regain full mobility in the knee joint, enhancing flexibility and reducing stiffness. Additionally, balance and proprioception exercises are crucial for improving joint awareness and stability, reducing the risk of re-injury. These goals together contribute to a comprehensive rehabilitation approach, promoting functional recovery and minimizing long-term complications.
	2. Improved Range of Motion: Work on achieving full knee flexion and extension as tolerated by your therapist.	
	3. Balance and proprioception: Exercises to enhance balance and spatial awareness in your knee joint.	
Precautions	1. Avoid downhill inclines: Walking or jogging downhill can place excessive stress on the knee joint, particularly after PCL reconstruction and ORIF for a patellar fracture.	Avoid downhill inclines to prevent excessive stress on the knee joint. Minimize activities involving rapid deceleration to avoid strain on reconstructed ligaments. Consider using a functional knee brace for added support during high-demand activities.
	2. Avoid activities with rapid deceleration: Activities that involve sudden stops or rapid changes in direction, combined with knee flexion, should be avoided.	
	3. Consider functional brace: During high-demand activities such as sports or heavy lifting, wearing a functional knee brace can provide additional support and stability.	
Intervention	1. Strengthening and endurance training-	It strengthens the quadriceps muscles, which are crucial for knee extension and walking. Progressive resistance increases muscle size and power output.
	Extension exercise in quadriceps chair:	
	Dosage: Perform 3 sets of 10 repetitions.	
	Progression: Increase weights gradually as tolerated for progressive resistance.	
	Hamstring strengthening:	Target hamstrings are important for hip flexion and knee stability. Increased resistance challenges these muscles for improved strength.
	Hamstring curls, Romanian deadlift.	
	Dosage: Perform 3 sets of 10 repetitions.	
	Progression: Increase resistance gradually as strength improves.	
	Squats and lunges with free weights:	Compound exercises work multiple muscle groups (quads, glutes, and hamstrings) for overall lower body strength and functional movement patterns. Progression ensures continued challenges for adaptation.
	Dosage: Perform 3 sets of 10 repetitions for each exercise.	
	Progression: Gradually increase weights or repetitions as strength and endurance improve.	
	Step up and step down:	
	Dosage: Perform 3 sets of 10 repetitions on each leg.	
	Progression: Increase the height of the step gradually for progressive difficulty.	
	Cycle ergometer:	Improves cardiovascular fitness (heart health and endurance) necessary for daily activities. Progression builds stamina for longer exertion.
	Dosage: Cycle for 15 minutes in both clockwise and anticlockwise directions.	
	Progression: Increase cycling duration or resistance as cardiovascular fitness improves.	
	Incline walking on treadmill:	It challenges the legs by adding an incline, mimicking walking uphill, and increasing cardiovascular demand. Progression builds strength and endurance for walking on varied terrain.
	Dosage: Walk on an incline for 15 minutes.	
	Progression: Gradually increase the incline or walking speed as tolerated.	
	Retro-walking on Treadmill:	Improves coordination, agility, and balance by challenging the nervous system to adapt to walking backwards. Progression enhances these skills for safer and more efficient movement.
	Dosage: Perform retro walking (walking backwards) on a treadmill for 15 minutes.	
	Progression: Increase walking speed or duration as coordination and agility improve.	
	2. Balance and proprioception:	It challenges balance by creating an unstable surface, activating core muscles, and improving overall stability. Adding arm movements and ball-catching further engages the nervous system for coordinated movement.
	Balancing in bilateral stance on wobble board:	
	Stand on the wobbleboard.	
	Balance for 30-60 seconds.	
	Repeat 2-3 sets.	
	Balance in bilateral stance on wobble board with arm movement and ball catching:	Standing on one leg strengthens that leg, while using resistance bands challenges core stability and coordination of the upper and lower body.
	Stand on the wobbleboard.	
	Perform arm movements.	
	Catch and throw the ball.	
	Complete 10 catches in each direction.	
	Repeat 2-3 sets.	
	Balancing in unilateral stance while performing upper extremity diagonal patterns with resistance bands:	Trains the body to maintain balance while reaching in different directions, improving proprioception (joint awareness) and stability in various directions.
	Stand on one leg.	
	Hold resistance bands.	
	Perform diagonal patterns.	
	Complete 10 repetitions on each side.	
	Repeat 2-3 sets.	
	Star excursion balance test as exercise:	
	Stand on one leg.	
	Reach in different directions.	
	Aim for 10 reaches in each direction.	
	Repeat 2-3 sets.	
Conclusion of rehabilitation	1. Absence of knee pain: Patients experience no discomfort in the knee joint during the final phase of rehabilitation, indicating effective pain management strategies and successful tissue healing.	Successful rehabilitation post-PCL reconstruction and ORIF for patellar fracture is characterized by the absence of knee pain and effusion, full restoration of knee mobility, attainment of 100% hamstring strength, and preservation of knee stability without instability, indicating optimal recovery and functional outcomes. Bottom of Form
	2. No joint effusion: swelling in the knee joint is notably reduced or absent, signifying resolution of inflammation and optimal joint health.	
	3. Restoration of knee mobility: Full active range of motion: Patients achieve complete knee flexibility, allowing for unrestricted movement and normal joint function.	
	4. Hamstring strength rehabilitation:	
	Attainment of 100% strength: Hamstring muscles on the surgical side match or exceed the strength of those on the unaffected side, enhancing knee stability and preventing imbalances.	
	5. Preservation of knee stability: Absence of instability: Patients do not report any instances of knee instability or giving way post-operatively, indicating successful restoration of joint stability and function.	

**Figure 4:**
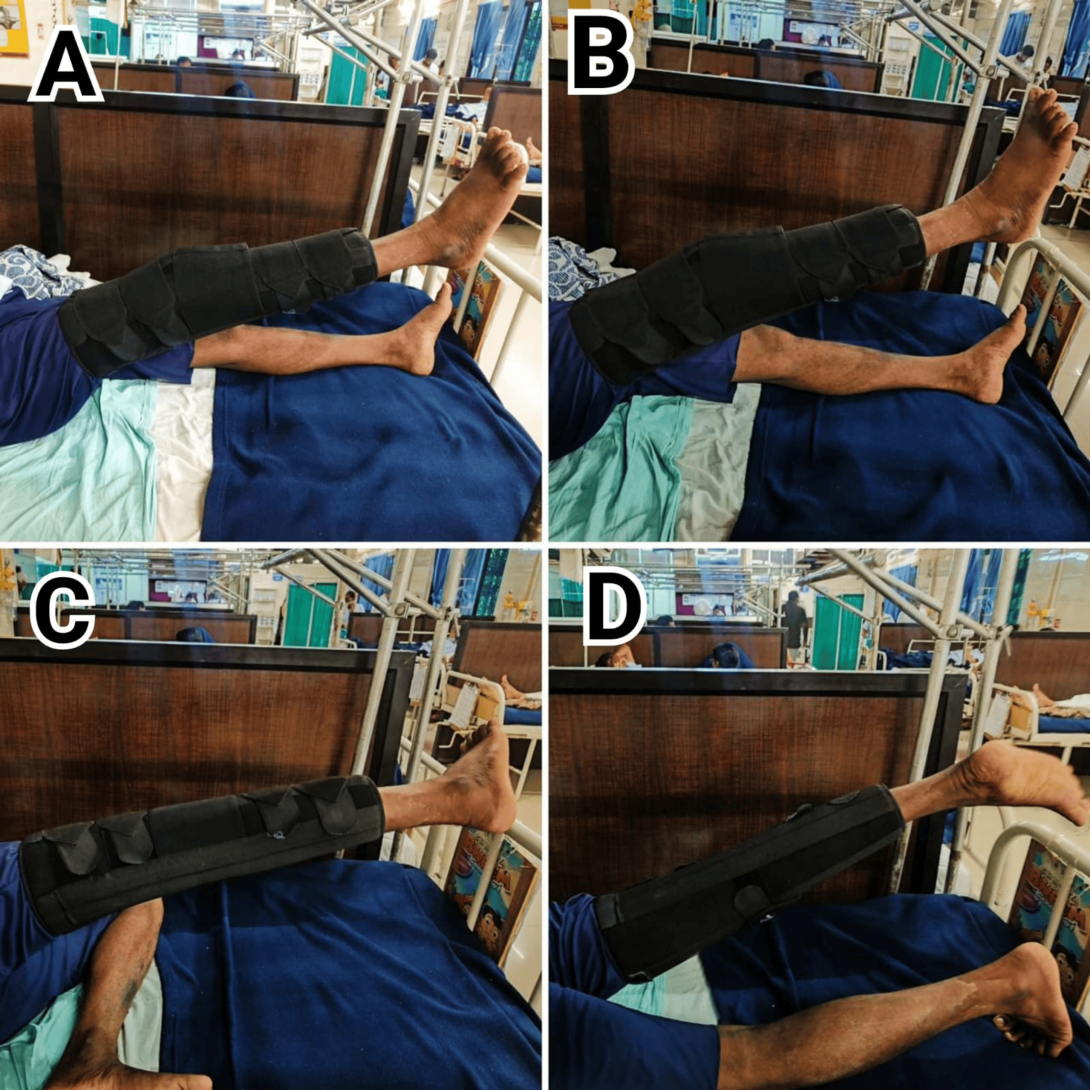
Physiotherapeutic intervention in phase 1 A: The patient is doing a straight leg raise; B: The patient is doing a multidirectional straight leg raise; C: The patient is doing a straight leg raise in a side-lying position for abductor strengthening; D: The patient is doing a straight leg raise in the prone position

Patient performing physiotherapy exercise in phase 1, shown in Figure 4.

Outcome measures

To assess your knee injury's impact on daily life, the outcome measures used are the KOOS questionnaire for pain, symptoms, activities, and quality of life; the TUG test to gauge basic mobility and balance; and the LEFS to evaluate your ability to perform tasks in your activities of daily living. For pain, the VAS is used. The interpretation of the values is given in Table 7.

**Table 7:** Outcome measures KOOS: Knee injury and osteoarthritis outcome score; TUG: Timed up and go; LEFS: Lower extremity functional scale N/A: Not assessable

Outcome measure	Pre-operative	Post-operative day 1	Post-rehabilitation week 6	Post-rehabilitation week 12
VAS	7.5	7.8	5.5	2
LEFS	1/80= 1.3%	2/80=2.5%	46/80= 57.6%	67/80= 83.85
KOOS	14%	13%	72%	84%
TUG	16.5 seconds	N/A	13 seconds	10 seconds

## Discussion

This case report outlines a comprehensive rehabilitation program, meticulously divided into distinct phases. This program prioritizes complete patient recovery, minimizes the risk of future complications, and allows a seamless return to their usual activities. By focusing on these objectives, the program aims to optimize the patient's long-term health and well-being. A male aged 44 years sustained a complex knee injury following a motorbike accident, resulting in a minimally displaced patellar fracture, a complete tear of the PCL, and a grade 2 tear of the posterior horn of the medial meniscus. The combination of a patellar fracture and a PCL avulsion fracture is notably rare, emphasizing the need to consider a wide range of potential injuries during the initial evaluation of knee trauma, particularly in high-impact scenarios [17]. Patellar fractures can obscure a concomitant PCL injury by complicating the performance of a posterior drawer test, making diagnostic imaging like MRI crucial for confirming the extent of ligament damage. The surgical intervention involved ORIF with a CC screw, a well-established method that provides stability and supports the healing of the PCL. After a comprehensive review of a case series by Rasmussen RG et al., the study demonstrates that patients with acute PCL injuries, treated with physiotherapy-led exercise and support brace intervention, experienced significant improvements in patient-reported outcomes and knee flexion strength over two years [18]. Also, according to Triska Monitari et al., exercise therapy post-acute PCL reconstruction offers benefits like maintaining patellar mobility, quadriceps muscle tone, full passive extension, pain and edema control, and early knee joint mobilization. Active movement training improves the range of motion and muscle elasticity and reduces pain, promoting peripheral circulation in the lower limbs. A structured and progressive rehabilitation program is essential for optimizing outcomes following PCL reconstruction and patellar fracture fixation. The rehabilitation protocol outlined in the case report focused on several key components, including pain management, swelling reduction, ROM restoration, muscle strengthening, neuromuscular control, and functional activities. To initiate early mobility, physiotherapy interventions, such as cryotherapy, ankle pumping exercises, and voluntary muscle activation, were employed to promote tissue healing and prevent joint stiffness. Additionally, a gradual progression of weight-bearing activities, balance exercises, proprioceptive drills, and cardiovascular conditioning was incorporated to enhance muscle strength, joint stability, and overall functional capacity [19].

The rehabilitation program led to favorable outcomes, as evidenced by the improvement in outcome measures over time. The patient experienced significant reductions in pain intensity, joint effusion, and functional impairment, with restored knee mobility, muscle strength, and stability [20]. The absence of knee pain, joint effusion, and instability indicated successful tissue healing, optimal joint function, and satisfactory recovery postoperatively. In conclusion, the comprehensive management approach described in these case reports underscores the importance of accurate diagnosis, appropriate surgical intervention, and structured rehabilitation in achieving favorable outcomes for patients with complex knee injuries involving patellar fractures and PCL tears. By addressing the unique challenges associated with each injury component and implementing a multidisciplinary treatment strategy, clinicians can optimize patient recovery and restore functional independence effectively.

While the case report demonstrates success, its generalizability might be limited due to the single-patient focus. Future studies with larger cohorts could validate the program's effectiveness and identify potential modifications for a broader application. Additionally, future research should focus on optimizing diagnosis, surgical techniques, rehabilitation protocols, and long-term outcomes for this specific fracture-ligament combination. This comprehensive approach, encompassing multiple phases of rehabilitation, aims to facilitate the patient's return to normal activities, minimize the risk of recurrent instability, and optimize long-term outcomes.

## Conclusions

This case report showcases the effective management of a rare combination of an undisplaced patellar fracture and a PCL avulsion fracture at the tibial attachment in a 44-year-old male patient. Successful treatment involved precise surgical fixation with a CC screw and a phased rehabilitation program. By week 12, the patient achieved significant improvements in pain, swelling, range of motion, and muscle strength, regaining near-normal knee function. This case emphasizes the importance of accurate diagnosis, appropriate surgical intervention, and individualized rehabilitation in managing complex knee injuries, highlighting the potential for excellent outcomes through tailored, holistic care.
